# Pseudomembranous Tracheobronchitis due to Mycobacterium tuberculosis

**DOI:** 10.7759/cureus.17173

**Published:** 2021-08-14

**Authors:** Luise J Froessl, Yazan Abdeen

**Affiliations:** 1 Medicine/Pulmonary, Baylor College of Medicine, Houston, USA; 2 Pulmonary and Critical Care Medicine, Pulmonary and Sleep Physicians of Houston, Webster, USA

**Keywords:** pseudomembranous tracheobronchitis, endobronchial tuberculosis, mycobacterium tuberculosis complex, tracheitis, endotracheal tuberculosis

## Abstract

Although the incidence is declining in the western world, *Mycobacterium tuberculosis* remains one of the most common infectious organisms responsible for significant morbidity and mortality worldwide. Pulmonary tuberculosis (TB) is the most commonly seen presentation; however, TB can affect nearly any of the body's organ systems. Endobronchial TB (EBTB) can complicate pulmonary TB or more rarely present as an isolated finding. It is essential to recognize and treat this condition promptly, as it can develop into tracheobronchial stenosis and chronic respiratory failure.

Here we discuss the case of a 43-year-old female with a left upper lobe cavitary lesion who presented with weight loss, dyspnea, and hoarseness. After failing multiple courses of antibiotics, the patient underwent bronchoscopy, and pseudomembranous tracheobronchitis (PMTB) was diagnosed. Cultures of endobronchial samples grew *Mycobacterium tuberculosis*. Standard anti-TB quadruple therapy was initiated, and the patient clinically improved.

Several subtypes of EBTB have been identified earlier. However, to the best of our knowledge, tuberculous PMTB has not previously been reported. This case allows us to consider the diagnostic and therapeutic implications of this condition.

## Introduction

Tuberculosis (TB) is one of the world’s most prevalent infectious diseases. Approximately 10 million people worldwide develop TB yearly [1]. In 1989, the Centers for Disease Control and Prevention (CDC) announced the objective of eliminating TB from the United States by 2010 [2]. Unfortunately, this goal was not met. This was due to several challenges including multidrug-resistant (MDR)-TB and sub-optimal clinical and public health management of the condition in certain countries. In 2019, about 2.7 cases of TB were reported per 100,000 people [3].

Of patients infected with *Mycobacterium tuberculosis*, about 90% will achieve either complete clearance of the infection or progress to a latent state. The remaining 10% will develop primary progressive disease [4]. The bacterium can cause a multiplicity of clinical manifestations and disseminate to almost any organ system in the human body. The most common presentation is pulmonary TB. Symptoms are generally nonspecific and can develop insidiously over several weeks or months. Patients can present with weight loss, fatigue, night sweats, and respiratory symptoms such as cough, sputum production, chest pain, and dyspnea [5].

Endobronchial TB (EBTB) is an uncommon presentation of TB. It results from the tuberculous infection of the trachea or bronchi [4]. The condition rarely presents with specific symptoms and can be challenging to diagnose. A significant complication is the development of tracheobronchial stenosis, which can occur in up to 90% of patients [6].

We report here a case of tuberculous pseudomembranous tracheobronchitis (PMTB) in an immunocompetent patient. To the best of our knowledge, no similar cases have previously been reported in the literature.

## Case presentation

A 43-year-old female with a prior medical history significant for hypertension and a 16 pack/year smoking history presented to the emergency department (ED) with progressive worsening of dyspnea and cough.

Symptom onset was two months ago. The patient reported being generally healthy with no known history of pulmonary disease. She noted a trip to Colorado as well as recent invasive dental work. During this period, the patient had been repeatedly diagnosed with bacterial pneumonia and was prescribed multiple courses of antibiotics, including a course of azithromycin, with no symptomatic improvement. A chest CT performed one month before presentation had shown a left upper lobe cavitary lesion.

On this occasion, she had been treated with a course of Augmentin, and an outpatient bronchoscopy had been ordered. However, due to the progression of symptoms, the patient was referred to the ED and subsequently hospitalized.

A review of symptoms on admission was positive for a cough productive of whitish sputum, exertional shortness of breath, hoarseness of the voice, and a weight loss of 20 pounds over two months. At the time of admission, the patient was afebrile with stable vital signs. Pertinent physical exam findings included stridor, bronchial breathing, and rales most prominent in the left upper lobe.

Initial laboratory workup showed evidence of anemia with a hemoglobin level of 7.5 g/dL, borderline leukocytosis, with a white blood cell count of 11.5 per microliter. A sepsis workup, auto-immune panel, and initial blood cultures were found to be negative. In addition, HIV testing was negative.

**Figure 1:**
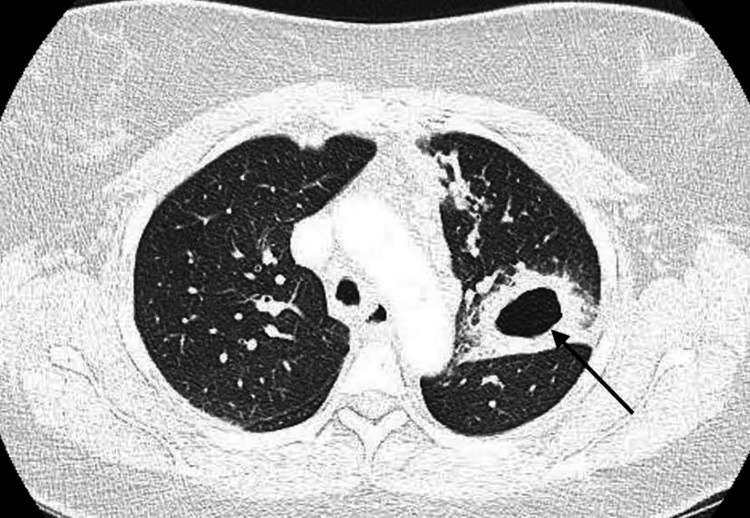
Computed tomography angiography showing the left upper lobe thick-walled cavity (arrow) 4.8 x 3.4 cm in diameter

CT of the chest was repeated and showed a left upper lobe cavitary lesion 4.4 x 3.4 cm in diameter (Figure 1). The lesion was shown to extend to the left hilum with a soft tissue density present, as well as mediastinal adenopathy and bilateral nodular infiltrates.

**Figure 2:**
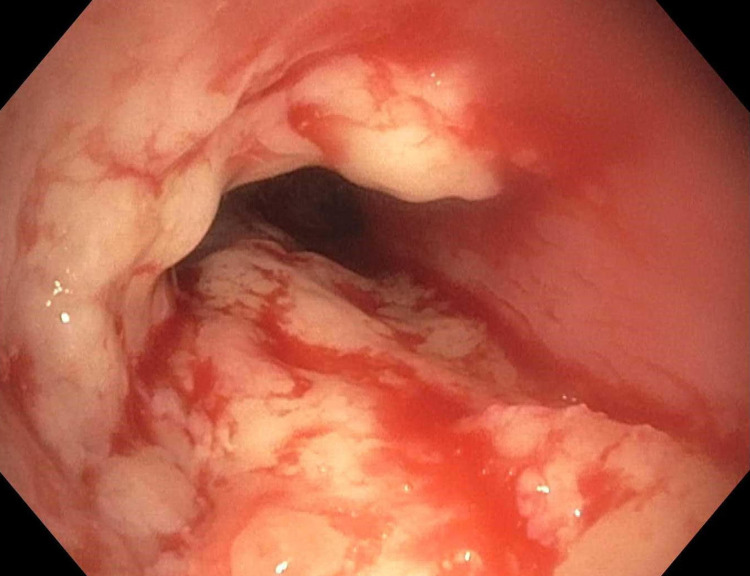
Endoscopic view of the upper third of the trachea showing pseudomembranous inflammation manifested by swelling, erythema, and cobble-stoning of the tracheal wall, along with thick turbid white secretions

The patient subsequently underwent bronchoscopy. Unexpectedly, evidence of pseudomembranous tracheitis was seen, with significant cobble-stoning, edema, and erythema of the entire trachea, along with a dynamic limitation of anteroposterior diameter (Figure 2).

**Figure 3:**
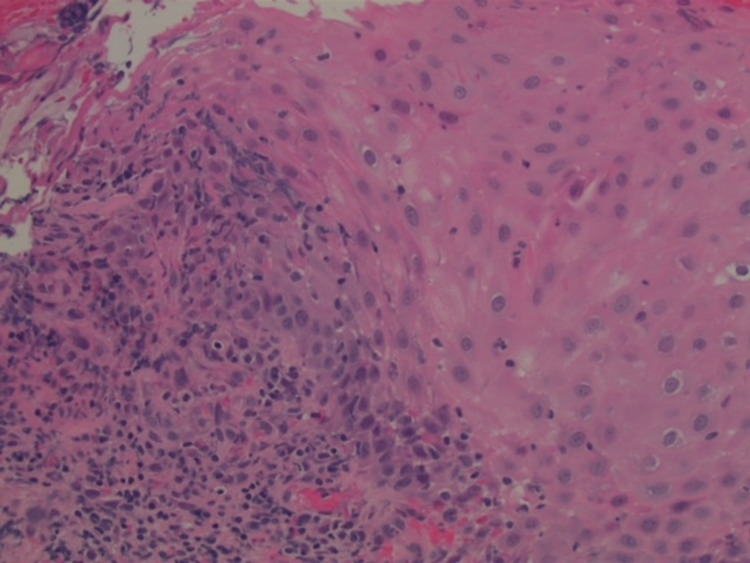
Inflammatory infiltrates visualized in the squamous mucosa of biopsy samples taken from the upper third of the trachea

Endobronchial biopsy of the trachea was performed, and histology showed ulceration, with prominent reactive acute and chronic inflammation (Figure 3).

No prominent eosinophils or definite granulomas were seen. Acid-fast, Alcian blue/periodic acid-Schiff, and Grocott's methenamine silver stains were performed and showed no acid-fast or fungal organisms. Endotracheal brushings and a left upper lobe bronchoalveolar lavage were performed. Additional biopsies were not collected, as the patient developed hypoxia during the procedure.

**Figure 4:**
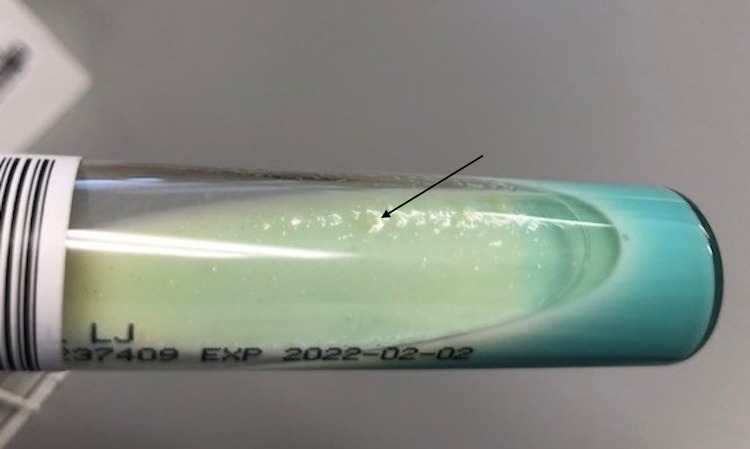
Brown, granular colonies of Mycobacterium tuberculosis (arrow) on the Löwenstein-Jensen medium

Of note, antifungals were added to the treatment regimen but were stopped due to negative cultures and lack of clinical response. The patient's respiratory condition stabilized, and she was discharged. However, the symptomatology of cough and dyspnea did not resolve completely despite antibiotic and antifungal therapies. Ultimately, cultures from samples collected during endotracheal brushing showed evidence of acid-fast bacillus growth consistent with tuberculosis (TB; Figure 4).

The patient was quickly contacted and started on an anti-TB quadruple regimen including isoniazid, rifampin, ethambutol, and pyrazinamide. Follow-up with the Department of Health for the duration of anti-TB treatment was established. The patient refused repeat bronchoscopy, but the clinical condition improved significantly with complete resolution of cough, dyspnea, and hoarseness.

## Discussion

PMTB is a rare condition generally encountered in patients predisposed to its development. The condition is characterized by an inflammation of the trachea and bronchial tree with the formation of pseudomembranes. The exact mechanisms remain unclear. It is generally accepted that an ischemic insult to the tracheobronchial wall is the initiating event. Ulceration and necrosis ultimately lead to the formation of a fibrinous exudate and a membrane-like structure that coats the trachea and bronchi [7].

Both infectious and non-infectious processes can cause PMTB. In patients with recent endotracheal intubation, the condition is likely related to endotracheal cuff pressure and ensuing damage to tracheal mucosa and submucosa. [7]. The most commonly reported infectious agent associated with PMTB is *Aspergillus fumigatus*. Other infectious etiologies, such as *Bacillus cereus* or *Pseudomonas aeruginosa*, have also been reported [8,9]. An underlying immune deficiency or recent viral infection is generally present in patients with infectious PMTB [10].

As mentioned, involvement of the trachea and bronchi in patients with pulmonary TB is frequent. It has been reported that more than half of patients may develop this complication [6,11]. Chung and Lee have classified EBTB into seven subtypes by bronchoscopic appearance [12]. However, the subtype of PMTB has not previously been reported. EBTB is more commonly seen in females and patients without a previous history of TB [11]. The patient presented here is female and, also, had no known history of TB at the time of presentation. Symptoms associated with EBTB include a barking cough, hemoptysis, sputum production, chest pain, or hoarseness [6]. Our patient presented with cough, sputum production, and hoarseness. It is plausible to consider that PMTB may be an additional subtype of EBTB that is presently underdiagnosed.

The diagnostic workup of pulmonary TB generally includes a chest X-ray and a follow-up chest CT [13]. Endobronchial or endotracheal TB is known to be a difficult diagnosis, as it does not present with specific symptoms or findings on chest X-rays, nor is systematically visualized on chest CT [14]. Bronchoscopy is not routinely performed on patients with pulmonary TB. A high index of clinical suspicion is therefore required [15]. Hoheisel et al. recommend performing bronchoscopy in all patients with pulmonary TB and evidence of volume loss on chest X-ray [16]. This approach is also ideal for identifying PTMB at the earliest possible stage.

The treatment of EBTB closely parallels that of pulmonary TB. The first-line treatment consists of the standard anti-TB quadruple therapy, with isoniazid, rifampicin, ethambutol, and pyrazinamide. The early initiation of treatment is essential to prevent the initiation of fibrosis and ultimately the development of stenosis [6]. Corticosteroids have been proposed to help prevent this complication; however, previous studies disagree on the efficacy [17,18]. Inflammation is known to play an essential role in PMTB. In the future, it may be of interest to explore the role of corticosteroids in the treatment of tuberculous PMTB.

Even with adequate treatment of EBTB, tracheobronchial stenosis is not always prevented [19]. The evolution of tuberculous PMTB is not well characterized at this time; however, it is not unlikely that it may also evolve to tracheobronchial stenosis. In addition, PMTB is known to cause acute upper airway obstruction, which can be life-threatening. Patients with tuberculous PMTB may therefore be at risk of both acute and chronic respiratory failure. This condition is essential to diagnose promptly and treat appropriately and effectively.

## Conclusions

In conclusion, tuberculous PMTB may be an underdiagnosed form of EBTB that is important to recognize early on in its evolution and treat adequately to prevent possible complications. The condition can present as a severe and acutely life-threatening upper airway obstruction. It is crucial for clinicians to be aware of this diagnosis and to include it in the differential for any patient with known TB infection presenting with acute respiratory distress.
